# Enhancing Sensitivity and Selectivity: Current Trends in Electrochemical Immunosensors for Organophosphate Analysis

**DOI:** 10.3390/bios14100496

**Published:** 2024-10-12

**Authors:** Yin Shen, Shichao Zhao, Fei Chen, Yanfei Lv, Li Fu

**Affiliations:** College of Materials and Environmental Engineering, Hangzhou Dianzi University, Hangzhou 310018, China; shenyin@hdu.edu.cn (Y.S.); zhaoshichao@hdu.edu.cn (S.Z.); feichen@hdu.edu.cn (F.C.); lvyanfei@hdu.edu.cn (Y.L.)

**Keywords:** nanomaterial-based amplification, microfluidic devices, aptasensors, signal transduction, environmental monitoring

## Abstract

This review examines recent advancements in electrochemical immunosensors for the detection of organophosphate pesticides, focusing on strategies to enhance sensitivity and selectivity. The widespread use of these pesticides has necessitated the development of rapid, accurate, and field-deployable detection methods. We discuss the fundamental principles of electrochemical immunosensors and explore innovative approaches to improve their performance. These include the utilization of nanomaterials such as metal nanoparticles, carbon nanotubes, and graphene for signal amplification; enzyme-based amplification strategies; and the design of three-dimensional electrode architectures. The integration of these sensors into microfluidic and lab-on-a-chip devices has enabled miniaturization and automation, while screen-printed and disposable electrodes have facilitated on-site testing. We analyze the challenges faced in real sample analysis, including matrix effects and the stability of biological recognition elements. Emerging trends such as the application of artificial intelligence for data interpretation and the development of aptamer-based sensors are highlighted. The review also considers the potential for commercialization and the hurdles that must be overcome for widespread adoption. Future research directions are identified, including the development of multi-analyte detection platforms and the integration of sensors with emerging technologies like the Internet of Things. This comprehensive overview provides insights into the current state of the field and outlines promising avenues for future development in organophosphate pesticide detection.

## 1. Introduction

Organophosphate pesticides (OPs) have been widely used in agriculture for decades due to their high efficacy in pest control [1]. However, the extensive application of these compounds has raised significant concerns regarding their impact on human health and the environment. OPs are known to inhibit acetylcholinesterase, a crucial enzyme in the nervous system, leading to potential neurotoxic effects in humans and wildlife [2,3]. The persistence of OPs in soil and water systems further exacerbates their environmental footprint, necessitating robust and sensitive detection methods to monitor and regulate their presence [4,5]. The development of highly sensitive and selective analytical techniques for OP detection has become a critical area of research in recent years. Traditional methods such as chromatography and mass spectrometry, while effective, often require complex sample preparation, expensive instrumentation, and specialized expertise [6,7,8]. These limitations have driven the search for more accessible, rapid, and field-deployable detection methods. In this context, electrochemical immunosensors have emerged as a promising alternative, offering advantages such as high sensitivity, real-time detection capabilities, and the potential for miniaturization and on-site analysis [9,10,11,12].

The detection of organophosphate pesticides has been a subject of intense research in recent years, given their widespread use and potential environmental and health impacts. Numerous reviews have explored various aspects of organophosphate detection, highlighting the diversity of approaches and technologies available. For instance, Kumaravel et al. [13] provided a comprehensive overview of different sensor types, including colorimetric, fluorescence, and electrochemical methods, emphasizing the advantages of nanomaterial integration in sensor performance. Kumaran et al. [2] expanded this perspective by discussing novel biosensing platforms such as organ-on-chip models, which offer insights into the effects of organophosphates on specific cellular systems. Bhattu et al. [5] focused on the development of portable sensors, comparing colorimetric, fluorescence, and electrochemical techniques for field applications. More specialized reviews and studies have also emerged, such as Chaudhari et al. [14] work on fiber-optic particle plasmon resonance biosensors, Arsawiset et al.’s [15] exploration of nanozyme paper-based analytical devices, Wang et al.’s development of a catalytic hairpin self-assembly-based fluorescent immunosensor, and Li et al.’s [16] innovative hydrogel test kit for on-site detection. While these reviews and studies have significantly contributed to our understanding of organophosphate detection methods, there remains a need for a focused examination of recent advancements in electrochemical immunosensors. This specific class of sensors offers unique advantages in terms of sensitivity, selectivity, and potential for miniaturization and field deployment. Electrochemical immunosensors combine the high specificity of antibody–antigen interactions with the sensitivity and simplicity of electrochemical detection methods, making them particularly well-suited for the challenges of organophosphate analysis in complex environmental and biological matrices.

**Figure 1:**
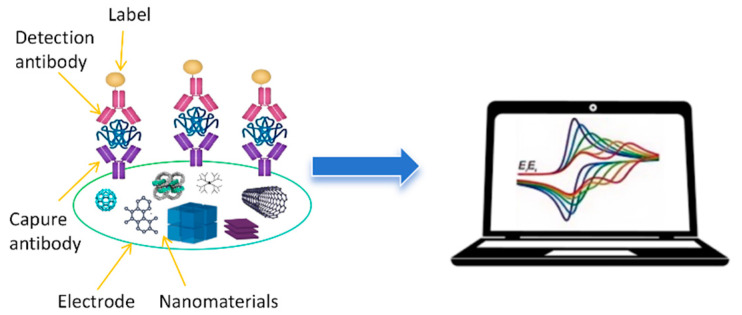
A schematic representation of an electrochemical immunosensor.

Electrochemical immunosensors utilize antibodies or antibody fragments, immobilized on an electrode surface, as recognition elements [17]. The binding of the target OP analyte to the antibody triggers an electrochemical signal, which can be measured and correlated with the concentration of the pesticide. The inherent amplification mechanisms in electrochemical reactions, coupled with the high specificity of antibody–antigen interactions, make these sensors particularly well-suited for trace-level detection of OPs in complex matrices. Figure 1 shows a scheme of a common electrochemical immunosensor for OP detection. Recent advancements in nanomaterials, surface chemistry, and fabrication techniques have significantly enhanced the performance of electrochemical immunosensors for OP detection. Researchers have explored various strategies to improve both the sensitivity and selectivity of these devices. Nanomaterial-based signal amplification, using substances such as metal nanoparticles and carbon nanomaterials, has proven effective in lowering detection limits [18,19]. Novel electrode modifications and enzyme-based amplification strategies have further pushed the boundaries of sensor sensitivity [20].

Electrochemical immunosensors can be broadly categorized into two main types: labeled and label-free. Labeled immunosensors utilize various signal-generating tags to amplify the detection signal [11]. These tags are typically attached to either the antigen or the detection antibody and can include enzymes, metal nanoparticles, or redox-active compounds. When the immunoreaction occurs, these labels generate a measurable electrochemical signal, often resulting in enhanced sensitivity. In contrast, label-free immunosensors directly measure changes in electrical properties upon antigen binding, without the need for additional labeling steps [21]. These sensors often rely on detecting alterations in electrode capacitance, charge transfer resistance, or other intrinsic electrical properties that occur when the target analyte binds to the immobilized antibodies. While label-free approaches offer simplicity and avoid potential interference from labels, they may sometimes suffer from limited sensitivity compared to their labeled counterparts. In the context of OP detection, both labeled and label-free electrochemical immunosensors have been explored, each finding specific applications based on the required sensitivity, sample complexity, and analytical setting.

The integration of electrochemical immunosensors into novel sensing platforms has opened up new possibilities for practical applications. Microfluidic and lab-on-a-chip devices offer the potential for automated [22,23], high-throughput analysis, while screen-printed and disposable electrodes provide cost-effective solutions for routine testing [24,25,26]. The development of wearable and flexible sensors points towards future applications in personal exposure monitoring and environmental surveillance [27,28]. Despite these advancements, several challenges remain in the widespread adoption of electrochemical immunosensors for OP detection. Issues such as sensor stability, reproducibility, and performance in complex real-world samples need to be addressed. Additionally, the path to commercialization and regulatory approval presents hurdles that must be overcome for these technologies to transition from the laboratory to practical field applications.

Our review aims to fill this gap by providing an in-depth analysis of the latest innovations in electrochemical immunosensors for organophosphate detection. We explore recent developments in electrode materials, nanomaterial-based signal amplification strategies, and novel sensing platforms specifically tailored for immunosensing applications. By focusing on this rapidly evolving subset of organophosphate detection technologies, we offer researchers and practitioners a comprehensive understanding of the current state of the art, persistent challenges, and promising future directions in the field. This targeted approach complements broader reviews by providing specialized insights into a technology with significant potential for advancing organophosphate monitoring in agriculture, environmental protection, and public health. Through this focused examination, we aim to stimulate further research and development in electrochemical immunosensors, ultimately contributing to more effective and accessible methods for organophosphate detection and analysis.

## 2. Fundamentals of Electrochemical Immunosensors for OP Detection

### 2.1. Principles of Electrochemical Detection

Electrochemical detection forms the cornerstone of immunosensors designed for OP analysis. This method relies on measuring electrical signals generated by redox reactions occurring at the electrode–solution interface. When OPs interact with the sensor surface, they induce changes in electrical properties such as current, potential, and impedance, which can be quantified to determine analyte concentration. The three primary types of electrochemical detection techniques employed in immunosensors are amperometry, voltammetry, and impedance spectroscopy. Amperometric detection involves applying a constant potential to the working electrode and measuring the resulting current, which is proportional to the analyte concentration. For example, Liu et al. [29] developed a label-free amperometric immunosensor for direct detection of paraoxon using a single-walled carbon nanotube (SWNT)-modified glassy carbon electrode (GCE). The amperometric immunosensor provided rapid, sensitive detection without the need for labeling, making it suitable for on-site monitoring of OPs. Voltammetric techniques, such as cyclic voltammetry and differential pulse voltammetry, involve scanning the potential over a defined range and recording the current response. For example, Dong et al. [30] developed an electrochemical immunosensor for detecting OPs using broad-spectrum antibodies and one-step electrodeposition. Gold nanoparticles were coupled with antibodies to form AuNP-Abs probes, which were then co-deposited with Prussian blue onto screen-printed carbon electrodes (SPEs). DPV was employed to measure the electrochemical response, providing a sensitive and selective detection method for OPs. The use of voltammetric techniques allowed for rapid and sensitive detection of OPs, with the ability to detect multiple pesticides simultaneously due to the broad-spectrum antibodies.

These methods provide rich information about redox processes and are particularly useful for multi-analyte detection. Electrochemical impedance spectroscopy (EIS) measures the impedance of the electrode system over a range of frequencies, offering insights into both Faradaic and non-Faradaic processes at the electrode surface. For example, Ding et al. [31] developed a portable pesticide residue detection instrument based on an impedance immunosensor. The immunosensor utilized novel multi-layer films of Au nanoparticles and polyaniline/carboxylated multi-wall carbon nanotube–chitosan nanocomposites. The detection principle relied on EIS to measure changes in impedance resulting from antigen–antibody interactions. The integrated system combined the immunosensor with a signal detection circuit for rapid on-site testing. Compared to existing portable devices using enzyme inhibition methods, this EIS-based instrument provided quantitative results more quickly and with less interference.

The specificity of electrochemical immunosensors for OP detection is derived from the highly selective interactions between antibodies and their target antigens. Antibodies are Y-shaped proteins produced by the immune system and have variable regions at their tips that recognize and bind to specific molecular structures called epitopes on antigen surfaces. In the context of OP detection, antibodies are typically raised against hapten–protein conjugates that mimic the structure of the target pesticide [32]. Antibody–antigen binding is governed by a combination of hydrogen bonding, van der Waals forces, and electrostatic interactions [33]. The strength of this interaction is characterized by the affinity constant, which plays a crucial role in determining the sensitivity and dynamic range of the immunosensor [34]. High-affinity antibodies are desirable for detecting low concentrations of OPs but may limit the sensor’s linear range.

### 2.2. Common Electrode Materials and Immobilization Strategies

The choice of electrode material and immobilization strategy significantly influences the performance of electrochemical immunosensors. Common electrode materials include gold and carbon materials. Each material offers unique properties in terms of conductivity, surface area, and compatibility with different immobilization techniques.

**Figure 2:**
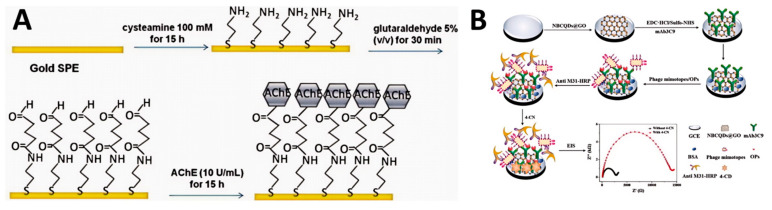
(**A**) Fabrication of Au-SPEs/SAM/AChE. Reprinted with permission from Ref. [35]. 2024, Elsevier. (**B**) GCE/NBCQDs@GO/mAb3C9 antibody/phage-mimotope M31/anti-M13 mAb-HRP. Reprinted with permission from Ref. [36]. 2024, Elsevier.

Gold electrodes are widely used due to their excellent conductivity and ease of surface modification through thiol chemistry. For example, Arduini et al. [35] developed an acetylcholinesterase (AChE) biosensor for detecting OPs based on enzyme inhibition. The researchers immobilized AChE on gold SPEs (Au-SPEs) using a self-assembled monolayer (SAM) of cross-linked cysteamine and glutaraldehyde (Figure 2A). Ferricyanide was used as an electrochemical mediator in solution to detect the enzymatic product thiocholine.

Carbon-based electrodes offer a wide potential window and low background current, making them suitable for sensitive detection. For example, Shi et al. [36] developed an electrochemical immunosensor for detecting O,O-dimethyl OPs using a phage mimotope and horseradish peroxidase (HRP). The GCE was modified with nitrogen and boron-doped carbon quantum dots and graphene oxide (NBCQDs@GO) to enhance conductivity and provide a large surface area. The sensor utilized a competitive binding mechanism between OPs and phage-mimotope M31 for antibody binding sites. HRP catalyzed the formation of insoluble precipitates, amplifying the impedance signal (Figure 2B).

Immobilization of antibodies on the electrode surface is a critical step in immunosensor fabrication. Common strategies include physical adsorption, covalent binding, and affinity-based immobilization. Physical adsorption is simple but may result in the random orientation and denaturation of antibodies [37]. Covalent binding, often achieved through carbodiimide chemistry or click reactions, offers more stable immobilization but can affect antibody activity if not carefully controlled [38]. Affinity-based methods, such as the biotin–streptavidin system [39] or protein A/G-mediated immobilization [40], allow for oriented antibody attachment, potentially improving sensor performance.

### 2.3. Signal Generation and Amplification Approaches

**Figure 3:**
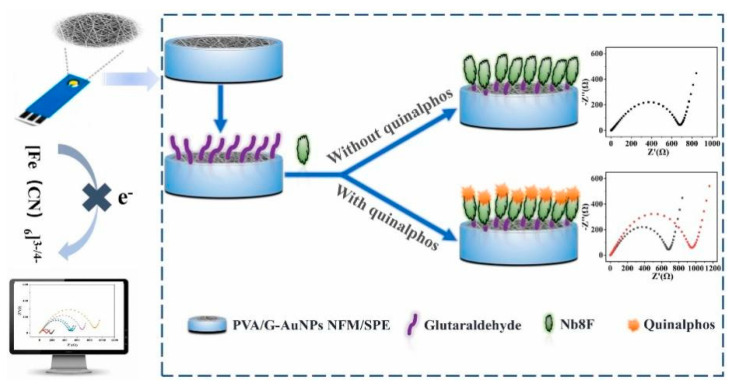
Scheme of fabrication procedure and sensing mechanism of cross-linked PVA/gelatin–AuNP NFM-based immunosensor for quinalphos detection. Reprinted with permission from Ref. [42]. 2024, Elsevier.

Signal generation in electrochemical immunosensors for OP detection can be broadly categorized into label-free and labeled approaches. Label-free methods rely on direct measurement of changes in electrical properties upon antigen binding, such as alterations in electrode capacitance or charge transfer resistance [41]. While these methods offer simplicity, they often suffer from limited sensitivity. For example, Hu et al. [42] developed a label-free electrochemical immunosensor for detecting quinalphos. The sensor was fabricated by immobilizing anti-quinalphos nanobodies directly onto a functionalized polyvinyl alcohol/gelatin–gold nanoparticle nanofiber membrane (PVA/G-AuNPs NFM) electrode (Figure 3). The label-free approach simplified the assembly process and avoided potential inactivation of binding sites. Under optimized conditions, the immunosensor exhibited a wide linear range (LDR) of 0.06–1000 ng/mL and a low detection limit (LOD) of 50.74 pg/mL for quinalphos. It demonstrated good specificity, with cross-reactivity below 12% for analogues. The sensor maintained over 90% activity after five regeneration cycles and about 90% activity after 6 weeks of storage. In spiked food samples, recoveries ranged from 89.68% to 110.88% with coefficients of variation of 1.65–7.81%. The results correlated well with UPLC-MS/MS analysis (R^2^ = 0.9859). The nanofiber membrane provided a large surface area for nanobody immobilization, with covalent binding increasing from 12.36 μg/cm^2^ to 40.07 μg/cm^2^ after glutaraldehyde activation. Wang et al. [37] developed a label-free impedimetric immunosensor for sensitive detection of fenvalerate in tea samples. They fabricated the sensor by modifying a GCE with chitosan and glutaraldehyde to immobilize fenvalerate antibodies. The label-free approach allowed for simple, rapid, and cost-effective detection without the need for expensive labeling reagents. Under optimized conditions, the sensor exhibited an LDR for fenvalerate concentrations from 1.0 × 10^−3^ to 1.0 × 10^−1^ mg/L, with an LOD of 0.8 μg/L. When applied to real tea samples, the method achieved an average recovery of 103%, with a 3.9% relative standard deviation, comparable to GC-MS results.

**Figure 4:**
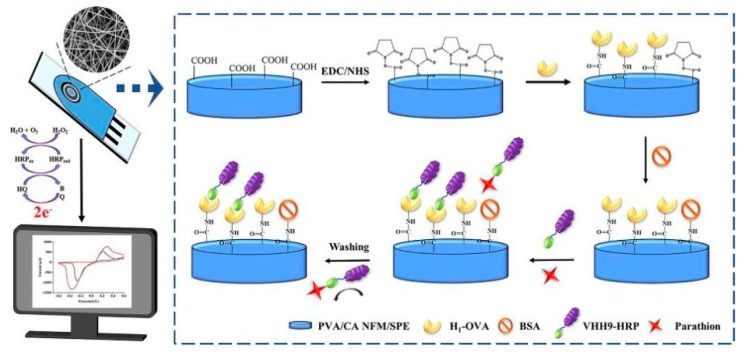
Scheme of fabrication procedure and sensing mechanism of cross-linked PVA/CA NFM-based immunosensor for parathion detection. Reprinted with permission from Ref. [43]. 2024, Elsevier.

Labeled approaches employ various signal-generating tags to amplify detection signals. Enzyme labels, such as horseradish peroxidase or alkaline phosphatase, catalyze redox reactions that generate measurable electrochemical signals. For example, Yin et al. [43] developed an electrochemical immunosensor for detecting parathion in food samples. The sensor utilized a cross-linked PVC/citric acid NFM (PVA/CA NFM)-modified SPE and a horseradish peroxidase (HRP)-labeled anti-parathion nanobody (Figure 4). The PVA/CA NFM provided an ideal microenvironment for biomolecule immobilization and facilitated rapid electron transfer. Under optimized conditions, the immunosensor exhibited excellent sensitivity, with a linear detection range of 0.01–100 ng/mL and a low detection limit of 2.26 pg/mL. It demonstrated good specificity against parathion analogs, with cross-reactivity below 11.31%. The sensor retained over 85% of its initial activity after 9 weeks of storage and could be regenerated up to three times while maintaining 85% performance. When applied to spiked food samples, the immunosensor achieved recoveries of 96.20–114.61% with coefficients of variation of 1.06–5.28%, correlating well with UPLC results (R^2^ = 0.9964). The use of nanobody labels enhanced the sensor’s stability and sensitivity compared to conventional antibodies. Similarly, Wang et al. [44] developed an immunochromatographic electrochemical biosensor (IEB) for rapid and sensitive detection of trichloropyridinol (TCP), a metabolite biomarker of exposure to OPs like chlorpyrifos (CPF). The IEB combined a lateral flow immunoassay strip with electrochemical detection using a screen-printed carbon electrode. HRP was used as an enzyme label to amplify the electrochemical signal. Under optimized conditions, the IEB achieved a wide linear range of 0.1–100 ng/mL and a low detection limit of 0.1 ng/mL for TCP. The total assay time was only 15 min. The IEB was successfully applied to detect TCP in rat plasma samples after in vivo exposure to chlorpyrifos-oxon. Compared to traditional ELISA, the IEB provided comparable results but with higher sensitivity, faster speed, and better portability. The use of the HRP enzyme label enabled sensitive electrochemical detection of the captured immunocomplexes. 

Nanoparticle labels, including metal nanoparticles and quantum dots, can enhance electron transfer or serve as carriers for multiple enzyme molecules, further amplifying the signal. For example, Lu et al. [45] developed a disposable electrochemical immunosensor for detecting organophosphorylated butyrylcholinesterase (OP-BChE), a biomarker for OP nerve agent exposure. The immunosensor utilized zirconia nanoparticles to capture OP-BChE and quantum dot-tagged antibodies for signal amplification. The use of nanoparticle labels significantly enhanced sensitivity, achieving a detection limit of 0.03 nM. The sensor demonstrated a linear response range from 0.1 to 30 nM OP-BChE with good reproducibility (4.5% relative standard deviation). Analysis of spiked human plasma samples yielded recoveries between 91.5% and 103.0%, indicating high accuracy and low matrix effects. The immunosensor was validated using in vitro exposure studies, showing a linear increase in OP-BChE adducts with increasing diisopropyl fluorophosphate concentrations up to 83 nM. This nanoparticle-based approach provided a simple, sensitive, and quantitative tool for rapid diagnosis of organophosphorus exposure, with potential applications in field testing and point-of-care (POC) diagnostics.

**Figure 5:**
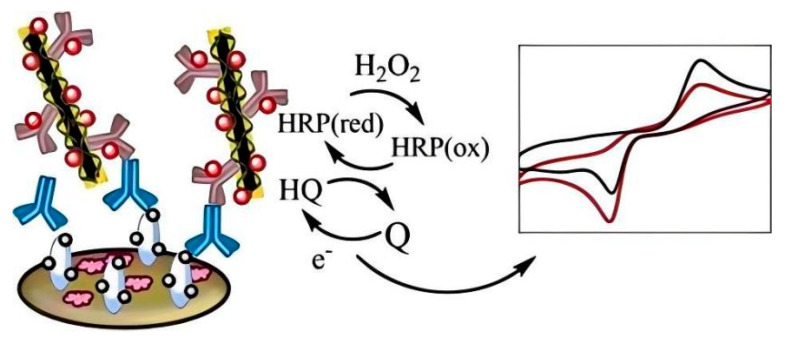
Scheme of electrochemical immunoassay for CPF detection. Reprinted with permission from Ref. [47]. 2024, Elsevier.

Signal amplification strategies play a crucial role in pushing the LOD of electrochemical immunosensors for OPs. Enzyme-based amplification cascades, where multiple enzymes work in tandem to generate a signal, have shown promising results [46]. Sun et al. [47] developed an electrochemical immunoassay for detecting CPF using a novel platform and signal amplification strategy. They created a three-dimensional sensor platform by modifying a GCE with quinone-rich polydopamine nanospheres. For signal amplification, they synthesized a multi-enzyme label consisting of HRP and secondary antibodies attached to flake-like Fe_3_O_4_-coated carbon nanotubes (Figure 5). This multi-enzyme label provided enhanced catalytic activity and a large surface area for immobilizing antibodies and enzymes. Under optimized conditions, the immunosensor exhibited an LDR of 0.01 to 1000 ng/mL and an LOD of 6.3 pg/mL for CPF. Compared to using only HRP-labeled secondary antibodies, the multi-enzyme label significantly improved sensitivity, expanding the LDR and lowering the LOD from 13.5 pg/mL to 6.3 pg/mL. The immunosensor showed good specificity; reproducibility, with coefficients of variation below 4.34%; and stability, retaining over 93% of the signal after 15 days of storage. When applied to spiked lake and pond water samples, it achieved recoveries between 96.7 and 110%, demonstrating its potential for sensitive CPF detection in real water samples. Nanomaterial-based amplification, using carbon nanotubes or graphene oxide as signal enhancers, for example, leverages the unique electronic properties of these materials to boost sensor performance [48,49,50]. 

### 2.4. Broader Applications of Electrochemical Immunosensing Principles

While this review focuses on organophosphate detection, it is important to recognize that the fundamental principles, strategies, and techniques discussed have broad applicability across the field of electrochemical immunosensing. The approaches developed for organophosphate detection can be adapted and applied to a wide range of target analytes, including other environmental contaminants, biomarkers, and food safety targets. The principles of electrochemical detection discussed in Section 2.1 are fundamental to all electrochemical immunosensors, regardless of the target analyte. For instance, the amperometric, voltammetric, and impedance spectroscopy techniques used in organophosphate detection are equally applicable to sensing other small molecules, proteins, and even whole cells. As an example, Carneiro et al. [51] applied similar electrochemical principles to develop a nanostructured label-free immunosensor for detecting α-synuclein, a biomarker for Parkinson’s disease.

The electrode materials and immobilization strategies outlined in Section 2.2 have broad relevance beyond organophosphate sensing. Gold electrodes and carbon-based materials, for instance, are widely used in electrochemical immunosensors for various targets. The covalent binding and affinity-based immobilization techniques discussed are applicable to a range of antibodies and recognition elements. For example, Boonkaew et al. [52] used similar immobilization strategies on paper-based electrodes to develop a cost-effective immunosensor for ferritin detection, demonstrating the versatility of these approaches. The signal generation and amplification approaches described in Section 2.3 are particularly transferable to other sensing applications. Enzyme labels like horseradish peroxidase and alkaline phosphatase are widely used across various electrochemical immunosensing platforms. Nanomaterial-based amplification strategies, such as the use of metal nanoparticles and carbon nanomaterials, have been successfully applied to enhance sensitivity in detecting numerous analytes. The challenges faced in real sample analysis, such as matrix effects and non-specific adsorption, are common across many areas of electrochemical immunosensing. Solutions developed for organophosphate detection, like the use of blocking agents or specialized sample preparation techniques, can often be adapted for other analytes. The emerging trends discussed, such as the integration of artificial intelligence for data analysis and the exploration of aptamer-based sensors, have potential applications far beyond organophosphate detection.

Novel sensing platforms like microfluidic devices and lab-on-a-chip systems, while discussed here in the context of organophosphate sensing, are being increasingly applied to a wide range of analytical challenges. For example, Surappa et al. [53] reviewed the application of similar microfluidic platforms for the isolation and analysis of cancer biomarkers, highlighting the broad utility of these approaches. By recognizing the wider applicability of these principles and techniques, researchers working on electrochemical immunosensors for various targets can draw valuable insights from the advancements made in organophosphate detection. This cross-pollination of ideas and approaches across different sensing applications has the potential to accelerate progress in the broader field of electrochemical immunosensing, leading to more sensitive, selective, and practical sensors for a wide range of analytical challenges.

In conclusion, electrochemical immunosensors offer several key advantages for organophosphate detection, including high sensitivity, selectivity, and the potential for rapid, on-site analysis. The combination of specific antibody–antigen interactions with sensitive electrochemical detection methods provides a powerful platform for quantifying these important environmental contaminants. The versatility in electrode materials, immobilization strategies, and signal amplification approaches allows for tailoring these sensors to meet the demanding requirements of organophosphate analysis in complex real-world samples.

## 3. Enhancing Sensitivity

### 3.1. Nanomaterial-Based Signal Amplification

Nanomaterials have revolutionized the field of electrochemical immunosensors, offering unprecedented opportunities for signal amplification and enhanced sensitivity in OP detection. The unique physical and chemical properties of nanomaterials, including high surface area-to-volume ratios, excellent conductivity, and catalytic activity, make them ideal candidates for improving sensor performance [48,49,50].

#### 3.1.1. Metal Nanoparticles

**Figure 6:**
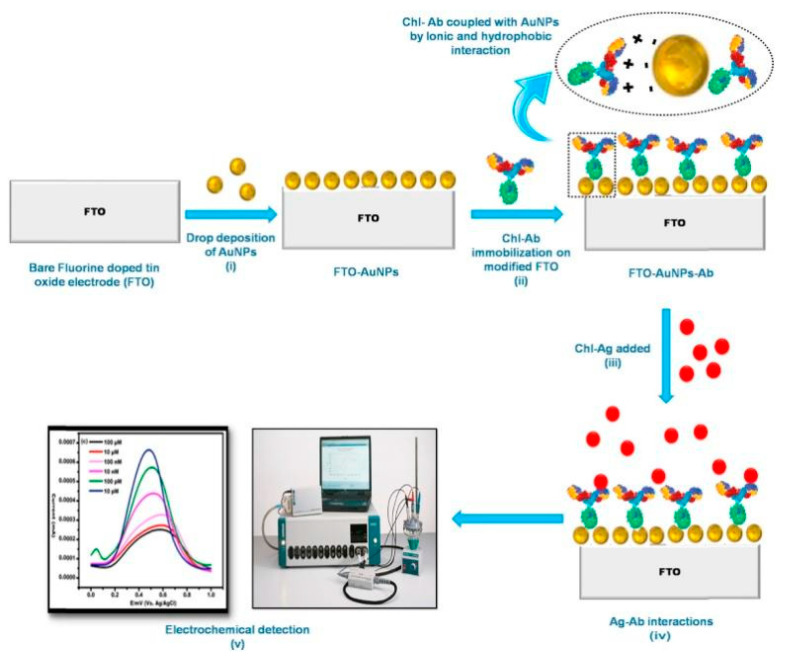
Scheme of the fabrication process of FTO-AuNPs-chl-Ab for CPF detection. Reprinted with permission from Ref. [56]. 2024, Elsevier.

Metal nanoparticles, particularly gold and silver, have been extensively employed in electrochemical immunosensors. These nanoparticles serve multiple roles, acting as electron transfer mediators, catalysts, and immobilization platforms for antibodies [54,55]. AuNPs are especially popular due to their biocompatibility and ease of functionalization. When incorporated into a sensor architecture, AuNPs can significantly enhance the electroactive surface area, facilitating faster electron transfer kinetics and resulting in amplified electrochemical signals. Talan et al. [56] developed an electrochemical nanosensor for detecting CPF using fluorine-doped tin oxide (FTO) electrodes modified with AuNPs and anti-CPF antibodies (Figure 6). AuNPs were utilized to enhance electrical conductivity and provide a platform for antibody immobilization, significantly improving the sensor’s sensitivity and performance. The fabricated FTO–AuNP–antibody nanosensor exhibited an LDR from 1 fM to 1 μM, with an impressive LOD of 10 fM. Cross-reactivity studies confirmed high specificity for CPF. The nanosensor successfully detected CPF in real food samples, including apples and cabbage at 10 nM and pomegranate at 50 nM. Dorozhko et al. [57] developed an electrochemical immunosensor for detecting carbaryl pesticide residues using copper nanoparticles (CuNPs) as labels. They synthesized a hapten–protein conjugate with CuNPs (Hap-Car-BSA@CuNPs) and used it in a direct solid-phase competitive assay. The CuNPs served as electrochemical labels, with the signal measured by linear sweep anodic stripping voltammetry on a gold–graphite electrode. The immunosensor demonstrated high sensitivity, with an LOD of 0.08 μg/kg and an LDR of 0.8–32.3 μg/kg in flour samples from different crops. Compared to conventional enzyme immunoassays, this CuNP-based approach proved cheaper, faster, and more user-friendly. The conjugate also exhibited greater stability than enzyme-labeled haptens. Recovery rates ranged from 93.1% to 97.5%, indicating minimal matrix interference and good accuracy. The intra-day coefficient of variation was below 8%, while the interday coefficient was under 12%, demonstrating good reproducibility.

Du et al. [58] developed an electrochemical immunosensor for detecting OP-AChE. The immunosensor utilized ZrO_2_ NPs to capture OP-AChE and lead phosphate–apoferritin-labeled anti-AChE antibodies (LPA-anti-AChE) for detection. ZrO_2_ NPs selectively bound phosphorylated AChE through metal chelation, overcoming the need for scarce phosphoserine-specific antibodies. Apoferritin nanoparticles encapsulating lead phosphate amplified the detection signal by encoding numerous metal ions. The sandwich immunoassay formed ZrO_2_/OP-AChE/LPA-anti-AChE complexes on SPEs. Under optimized conditions, the immunosensor exhibited a linear response from 0.05 to 10 nM OP-AChE, with a 0.02 nM LOD. It demonstrated good reproducibility with intra- and inter-assay coefficients of variation below 6%. The method achieved 96.4–106.7% recovery when detecting OP-AChE spiked in rat plasma samples. By combining ZrO_2_ NPs and apoferritin-templated metallic tags, this simple, sensitive approach enabled quantitative monitoring of OP exposure without requiring OP-specific antibodies, offering advantages in selectivity, sensitivity, and potential field applications. Wang et al. [59] developed a Co_3_O_4_/polyaniline (PAn) magnetic nanoparticle-modified electrochemical immunosensor for rapid detection of CPF residues in agricultural products. The researchers utilized Co_3_O_4_ nanoparticles coated with PAn to improve electrode conductivity and dynamic performance. The sensor demonstrated high sensitivity and accuracy, with an LDR of 0–10 μg/mL and an LOD of 0.01 μg/mL. Recovery rates in apple and vegetable samples exceeded 82%, with coefficients of variation below 5%. The immunosensor could be regenerated using glycine–HCl buffer while maintaining reliable sensitivity. Co_3_O_4_/PAn nanocomposites, with particle sizes around 80 nm, enhanced the electrode’s conductivity and sensing capabilities. The use of silver nanoparticle-labeled secondary antibodies further amplified the signal, improving sensitivity compared to traditional single-label methods.

**Figure 7:**
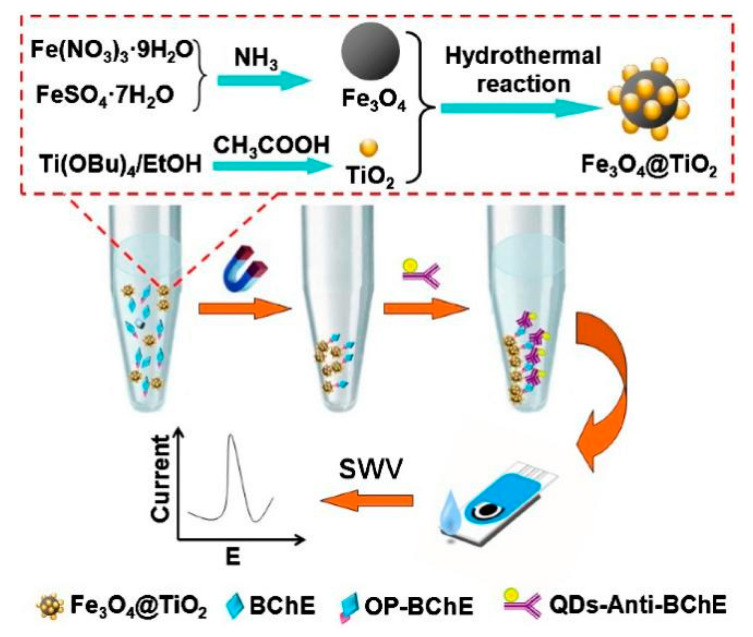
Scheme of the fabrication process of Fe_3_O_4_ at TiO_2_ magnetic nanoparticles for BChE detection. Reprinted with permission from Ref. [60]. 2024, Elsevier.

Recent advancements in metal nanoparticle-based signal amplification include the development of bimetallic nanoparticles. For instance, Zhang et al. [60] developed an immunosensor for detecting OP-BChE. They synthesized Fe_3_O_4_@TiO_2_ magnetic nanoparticles to selectively capture phosphorylated proteins, overcoming the limitation of scarce OP-specific antibodies. These nanoparticles served as capture agents in a sandwich immunoassay, with quantum dot-tagged anti-BChE antibodies for secondary recognition. The magnetic properties allowed easy separation from complex biological matrices (Figure 7). The immunosensor exhibited a linear response range of 0.02–10 nM OP-BChE, with a detection limit of 0.01 nM. This sensitivity was comparable to mass spectrometric methods, capable of detecting less than 1% BChE inhibition. The method showed good reproducibility, with intra-assay coefficients of variation below 6.2% and inter-assay coefficients below 6.7%. When tested with spiked human plasma samples, recoveries ranged from 92 to 105%, demonstrating reliability in complex matrices. The Fe_3_O_4_@TiO_2_ nanoparticles effectively combined selective phosphoprotein enrichment with magnetic separation, enabling sensitive and specific detection of OP exposure biomarkers in a portable, inexpensive format.

#### 3.1.2. Carbon Nanomaterials

**Figure 8:**
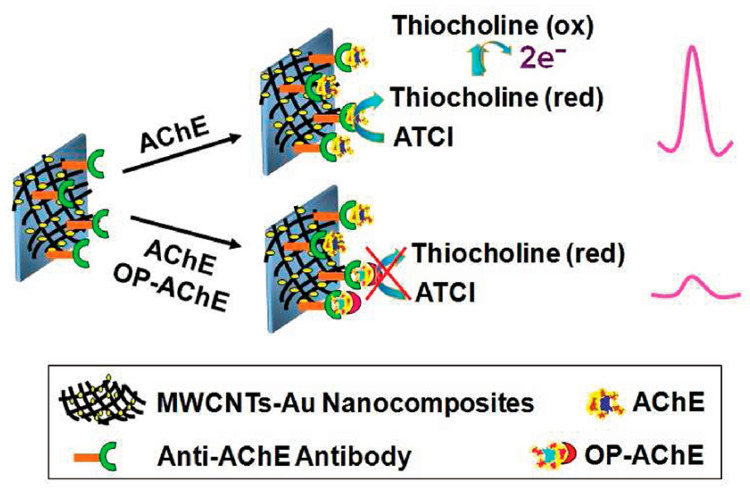
Scheme of immunosensing platform for measurement of enzyme activity and OP exposure. Reprinted with permission from Ref. [63]. 2024, ACS.

Carbon nanomaterials, including carbon nanotubes (CNTs), graphene, biochar [61], and carbon dots, have emerged as powerful tools for signal amplification in OP detection. These materials offer exceptional electronic properties, high surface areas, and versatile functionalization options. Multi-walled carbon nanotubes (MWCNTs) and SWCNTs have been extensively used to modify electrode surfaces, creating three-dimensional networks that facilitate rapid electron transfer and provide numerous binding sites for antibody immobilization. For example, a multi-analyte electrochemical immunosensor was developed for detecting pesticides like endosulfan and paraoxon using SWCNTs patterned on GCE [62]. This approach utilized aryldiazonium salt chemistry for covalent attachment, enabling efficient electron transfer between biomolecules and electrodes. The immunosensor demonstrated high sensitivity and selectivity, with LODs of 0.05 ppb for endosulfan and 2 ppb for paraoxon. The SWCNTs provided a stable interface, enhancing the sensor’s performance and allowing simultaneous detection of multiple analytes. The immunosensor exhibited linear responses over ranges of 0.05–100 ppb for endosulfan and 2–2500 ppb for paraoxon, making it suitable for environmental monitoring. The research highlighted the potential for using SWCNTs in portable devices for on-site pesticide detection, emphasizing the importance of stable and efficient interfaces in biosensor design. Chen et al. [63] developed a method for AChE activity assay to monitor exposure to OPs. The approach utilized selective immunocapture of AChE followed by electrochemical detection of enzyme activity. A disposable electrochemical sensor based on MWCNT-Au nanocomposites was used to immobilize AChE-specific antibodies (Figure 8). MWCNT-Au nanocomposites greatly enhanced electron transfer and electrocatalytic oxidation of thiocholine, improving sensitivity. The method demonstrated a linear response for AChE concentrations from 0.1 to 10 nM, with an LOD of 0.05 nM. For paraoxon-dosed AChE solutions, inhibition was proportional to the paraoxon concentration from 0.2 to 50 nM. The technique was validated using in vitro paraoxon-exposed red blood cell samples, detecting less than 5% AChE inhibition.

**Figure 9:**
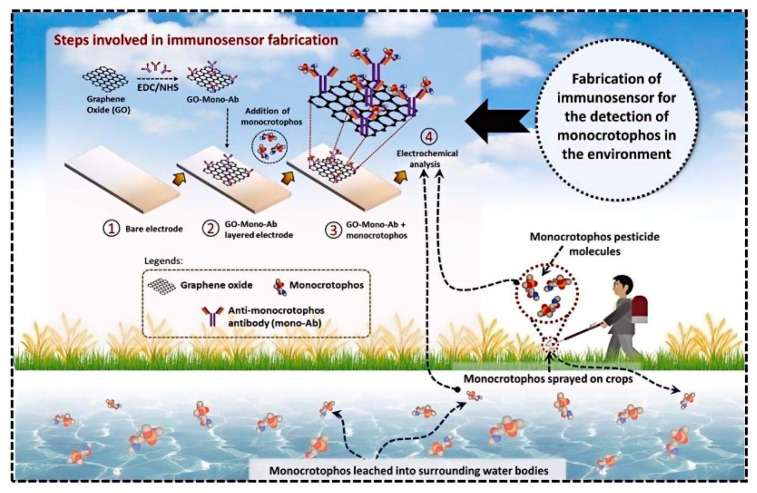
Detection of monocrotophos using an immunosensor with randomly layered GO. Reprinted with permission from Ref. [69]. 2024, Elsevier.

Graphene and its derivatives, such as reduced graphene oxide (rGO), have gained significant attention due to their unique two-dimensional structure and excellent conductivity. These materials can be easily functionalized and integrated into various sensor architectures, serving as both signal amplifiers and carriers for other nanomaterials or biomolecules [64,65,66]. The incorporation of graphene-based materials has enabled the development of ultrasensitive immunosensors capable of detecting OPs at sub-picomolar levels. For example, Metha et al. [67] developed a graphene-modified SPE immunosensor for the sensitive detection of parathion. The sensor was fabricated by modifying SPEs with graphene sheets, which were then functionalized with 2-aminobenzyl amine. This modification allowed the attachment of anti-parathion antibodies, creating a biosensor capable of detecting parathion through electrochemical impedance spectroscopy. The sensor demonstrated an LDR of 0.1–1000 ng/L and an LOD of 52 pg/L. It also showed high selectivity for parathion even in the presence of other pesticides like malathion, paraoxon, and fenitrothion. The use of graphene significantly enhanced the sensor’s sensitivity and selectivity due to its high surface area and excellent electrical properties. In another work [68], the authors focused on the development of a graphene QD (GQD)-modified SPE immunosensor for detecting parathion. GQDs were utilized due to their excellent electron-accepting and -donating capabilities, high stability, low toxicity, and ease of functionalization. The immunosensor demonstrated a dynamic linear response to parathion concentrations ranging from 0.1 to 10^6^ ng/L, with an LOD of 46 pg/L. Shrikrishna et al. [69] focused on developing an electrochemical immunosensor for detecting monocrotophos. They designed the sensor using antibodies specific to monocrotophos conjugated with graphene oxide (GO) and layered onto a fluorine-doped tin oxide (FTO) electrode (Figure 9). This setup enhanced the sensor’s performance, achieving a detection limit of 0.49 ppm. GO was utilized as an electrochemical mediator, improving electron transfer efficiency and stability. The sensor demonstrated specificity for monocrotophos, even in the presence of other pesticides like malathion and methidathion. It also maintained stability for up to four weeks. 

Biochar has gained attention as a cost-effective and eco-friendly material that can serve as both an electrochemical enhancer and an effective substrate for immobilizing bioreceptors. For example, Cancelliere et al. [70] developed a label-free electrochemical immunosensor for detecting Interleukin-6 (IL-6) in serum samples from psoriasis patients. It utilized a sandwich-based format with two primary antibodies and screen-printed electrodes modified with biochar. Biochar served both as an electrochemical enhancer and an anchoring system for bioreceptor immobilization. The immunosensor demonstrated robust analytical performance, with a wide linear detection range from 2 to 250 pg/mL and an LOD of 0.78 pg/mL. It showed excellent reproducibility, with an RSD of less than 7%. Validation with an ELISA kit on 25 serum samples confirmed strong correlation in IL-6 concentration measurements, highlighting the device’s ease of use and rapid detection capabilities.

### 3.2. Enzyme-Based Amplification Strategies

Enzyme-based amplification strategies continue to play a crucial role in enhancing the sensitivity of electrochemical immunosensors for OP detection. These approaches leverage the catalytic activity of enzymes to generate or amplify electrochemical signals. Common enzymes used in these systems include HRP and glucose oxidase (GOx). For example, a sensitive electrochemical impedimetric immunoassay was developed for the determination of CPF using a nanogold-modified GCE [71]. The assay utilized HRP conjugated with AuNPs and bovine serum albumin–CPF (BSA-CPF) as an analyte competitor. HRP played a crucial role in signal amplification through a biocatalytic precipitation process, which produced an insoluble compound that increased the Faradaic impedance of the electrode. The immunoassay exhibited a linear detection range between 0.001 ng/mL and 10 ng/mL, with a detection limit of 0.070 pg/mL. The method was tested on artificially spiked vegetables, achieving recovery rates between 85% and 110% and relative standard deviations below 7.5%. The use of HRP and AuNPs enhanced the sensitivity and specificity of the assay, making it a promising tool for detecting low concentrations of CPF in complex matrices like food and environmental samples. Keay and McNeil [72] reported a separation-free electrochemical immunosensor for the rapid detection of the pesticide atrazine in water samples. This innovative method employed a competitive ELISA format, integrating disposable SPE modified with HRP and single-use atrazine immunomembranes. The SPEs were created using carbon ink infused with HRP, and monoclonal antibodies specific to atrazine were immobilized on Biodyne C membranes, which were then placed over the electrode surface. The assay operated on a principle where free atrazine and an atrazine–GOx conjugate competed for binding sites. In the presence of glucose, the GOx produced hydrogen peroxide, which was reduced via enzyme channeling at the HRP electrode, allowing for direct electron transfer at a potential of +50 mV versus Ag/AgCl. The system showed excellent intra-electrode reproducibility, with a coefficient of variation of less than 4% and a detection limit of 0.012 mg/L, which was significantly lower than the maximum admissible concentration of 0.1 mg/L set by the European Community. Field tests in Israel using river water samples spiked with atrazine revealed recovery rates between 98% and 101%, indicating minimal matrix effects.

### 3.3. Three-Dimensional Electrode Architectures

**Figure 10:**
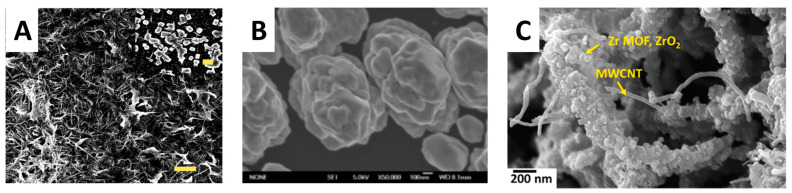
SEM image of (**A**) MOF/ITO (porous morphology). Reprinted with permission from Ref. [73]. 2024, Elsevier. (**B**) three-dimensional gold nanoclusters. Reprinted with permission from Ref. [74]. 2024, Elsevier. (**C**) Zr-MOF/ZrO_2_/MWCNT ternary composite.

Innovative electrode modifications have emerged as a key strategy for enhancing electron transfer and improving the sensitivity of electrochemical immunosensors. These modifications aim to increase the electroactive surface area, facilitate charge transfer, and provide a suitable environment for antibody immobilization. Three-dimensional electrode architectures, such as nanostructured metal oxides, conducting polymer networks, and hierarchical carbon structures, have shown great promise in improving sensor performance. These structures offer increased surface areas for antibody immobilization and enhanced mass transport of analytes to the electrode surface. For example, Chansi et al. [73] developed an electrochemical immunosensor utilizing a layered construction of a nanoimmunohybrid embedded in a metal–organic framework (MOF) to detect total pesticide loads in vegetable extracts. The sensor, comprising a BSA/Chi-AuNP-rIgG-BSA/MOF/ITO platform, effectively integrated polyclonal antibodies with MOF-modified ITO substrates. The 3D porous morphology of MOF-5 enhanced the sensor’s performance by providing a large surface area and abundant functional groups for pesticide screening (Figure 10A). This design facilitated high sensitivity and selectivity, achieving a linear detection range from 4 to 100 ng/L. The sensor exhibited a rapid analysis time of just one minute, maintaining stability for up to 25 days. Chen et al. [74] reported an electrochemical immunosensor for detecting trace amounts of picloram using three-dimensional Au nanoclusters. The three-dimensional Au nanoclusters were synthesized through a two-step electrodeposition process, which enhanced the sensor’s performance by providing a large surface area and facilitating electron transfer (morphology shown in Figure 10B). This method allowed for the immobilization of BSA–picloram and subsequent competitive immunoreaction with picloram antibodies. The sensor demonstrated a broad LDR from 0.001 to 10 µg/mL, with a correlation coefficient of 0.996. It achieved an LOD of 0.0005 µg/mL. The innovative use of 3D Au nanoclusters significantly contributed to the sensor’s high sensitivity and wide linear range, proving its potential for practical environmental monitoring of picloram. Rahmani et al. [75] developed an electrochemical sensor for detecting carbaryl using a modified three-dimensional graphene-gold (3DG-Au) nanocomposite. The 3DG was synthesized with thiourea, introducing sulfur and nitrogen functional groups, and subsequently modified with gold nanoparticles to enhance its electrochemical properties. The sensor demonstrated a linear detection range for carbaryl from 0.004 to 0.3 μM with an impressive detection limit of 0.0012 μM. Gokila et al. [76] developed a non-enzymatic electrochemical impedance sensor using a ternary composite of Zr-MOF/ZrO_2_/MWCNT (Figure 10C) for the selective detection of electro-inactive OPs. The ternary composite was synthesized via a solvothermal process, showing an enhanced surface area of 1158 m^2^/g compared to the pristine Zr-MOF’s 868 m^2^/g. The sensor demonstrated high selectivity and sensitivity towards OPs containing both phosphorus and sulfur, with detection limits of 2.02 nM for malathion, 2.8 nM for chlorpyrifos, 2.5 nM for dimethoate, 1.11 nM for monocrotophos, and 2.01 nM for glyphosate.

The various strategies discussed for enhancing sensitivity, from nanomaterial-based amplification to innovative electrode architectures, demonstrate the significant progress made in pushing the detection limits of electrochemical immunosensors for organophosphates. These advancements are crucial for meeting the increasingly stringent regulatory requirements and for enabling the detection of trace levels of these compounds in environmental and biological matrices. The improved sensitivity offered by these approaches positions electrochemical immunosensors as highly competitive tools for organophosphate monitoring compared to traditional analytical methods.

## 4. Novel Sensing Platforms and Fabrication Methods

The development of novel sensing platforms and fabrication methods has significantly advanced the field of electrochemical immunosensors for organophosphate detection. These innovations have not only enhanced sensor performance but also opened up new possibilities for on-site, real-time monitoring of these pesticides in various environmental and biological matrices.

### 4.1. Microfluidic and Lab-on-a-Chip Devices

Microfluidic and lab-on-a-chip devices represent a major leap forward in the miniaturization and integration of electrochemical immunosensors [77]. These platforms offer numerous advantages, including reduced sample and reagent consumption, faster analysis times, and the potential for automation and high-throughput screening [56,78,79]. In the context of OP detection, microfluidic devices have enabled the development of compact, portable systems capable of performing complex analytical procedures in field settings.

**Figure 11:**
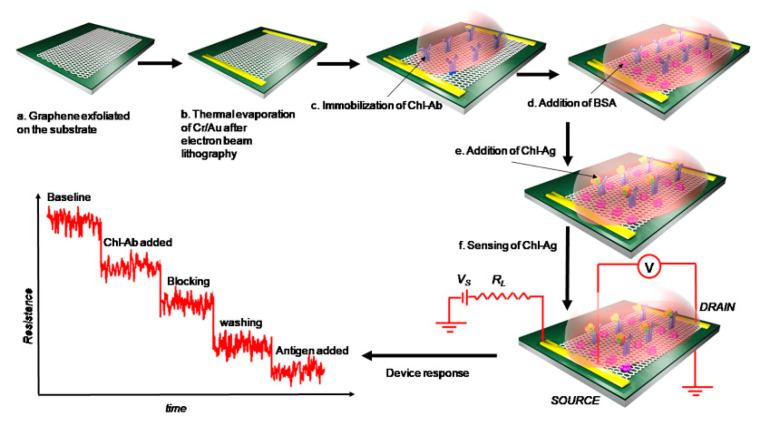
Fabrication procedure for graphene FET device for CPF detection. Reprinted with permission from Ref. [81]. 2024, Springer.

Jia et al. [80] reported an electrochemical immunosensor integrated with a microfluidic chip for the rapid detection of CPF. This innovative device utilized a microfluidic chip with embedded gold interdigitated array microelectrodes (IDAMs), which were modified using nanomaterials and protein A to bind antibodies. The binding of CPF to the antibody-modified IDAMs resulted in a detectable change in impedance, measured through EIS. The microfluidic chip demonstrated several advantages, including an LDR from 0.5 to 500 ng/mL, with an LOD of 0.5 ng/mL. Islam et al. [81] developed a microfluidic-based graphene field-effect transistor (graFET) for the sensitive detection of CPF (Figure 11). The study aimed to create a robust immunobiosensor for real samples, utilizing a graFET fabricated on a Si/SiO_2_ substrate. Anti-CPF antibodies were immobilized on the graphene surface, allowing the sensor to detect CPF over an LDR from 1 fM to 1 µM, with an LOD of 1.8 fM. The graFET biosensor demonstrated its potential for on-site applications and could be integrated into electronic chips for detecting CPF and other OPs in food and environmental samples.

In the field of microfluidic devices for organophosphate detection, the choice of materials plays a crucial role in determining the performance, cost-effectiveness, and applicability of sensors. Polydimethylsiloxane (PDMS) has emerged as a widely used material for fabricating microfluidic channels, offering several advantages that make it particularly suitable for this application. The optical transparency of PDMS allows for easy visual inspection and integration with optical detection methods, which is essential for many immunoassay-based sensors. Its biocompatibility ensures that it does not interfere with biological components such as antibodies or enzymes used in the sensing mechanism. Furthermore, the ease of molding PDMS enables rapid prototyping and cost-effective production of complex microfluidic structures, facilitating the development of intricate channel designs for efficient sample handling and analyte detection. Glass substrates have also maintained their importance in microfluidic immunosensor fabrication, particularly when dealing with organophosphate pesticides. The excellent chemical resistance of glass makes it ideal for applications involving organic solvents or harsh cleaning procedures, which are often necessary when working with environmental samples potentially contaminated with pesticides. In recent years, there has been a growing interest in paper-based microfluidic devices, especially in resource-limited settings or for rapid on-site testing. These devices leverage the capillary action of paper to move fluids without external pumps, significantly reducing complexity and cost. The disposable nature of paper-based devices addresses concerns about cross-contamination between samples, which is particularly important when dealing with persistent organic pollutants like organophosphates.

### 4.2. Screen-Printed and Disposable Electrodes

**Figure 12:**
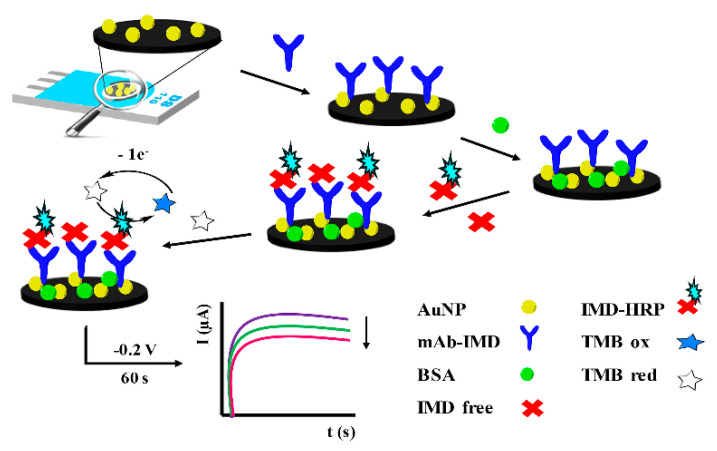
Scheme of the direct competitive immunosensor for the detection of IMD on AuNP-SPEs using monoclonal antibodies. Reprinted with permission from Ref. [85]. 2024, Elsevier.

SPEs have emerged as a cost-effective and versatile platform for the development of electrochemical immunosensors for organophosphate detection. These electrodes are fabricated by printing conductive inks onto various substrates, allowing for mass production and customization of electrode designs [82,83]. The disposable nature of SPEs addresses concerns about electrode fouling and cross-contamination, making them particularly suitable for on-site environmental monitoring and POC diagnostics [84]. Pérez-Fernández et al. [85] developed a direct competitive immunosensor for detecting imidacloprid (IMD) using AuNP-modified SPEs (AuNP-SPEs). This innovative approach utilized monoclonal antibodies immobilized on the modified electrodes, allowing free IMD in samples to compete with enzyme-labeled IMD for antibody recognition (Figure 12). The sensor exhibited an impressive analytical performance, with an LOD of 22 pM, which is below the regulatory limits. It demonstrated an LDR from 50 to 10,000 pM, high precision with a relative standard deviation of 6%, and good accuracy with a relative error of 6%. The use of AuNP-SPEs was pivotal, enhancing the electroactive surface area and facilitating electron transfer, thus improving sensitivity and selectivity. El-Moghazy et al. [86] developed a nanobody-based electrochemical immunosensor utilizing decorated nylon nanofibers for POC monitoring of human exposure to pyrethroid insecticides. This sensor specifically targeted 3-Phenoxybenzoic acid (3-PBA), a common biomarker for pyrethroid exposure, by leveraging an SPE as a platform. The SPE was chosen for its ability to enable rapid, real-time detection with high sensitivity and selectivity due to the specific antibody–antigen interactions on its surface. The sensor demonstrated an LDR of 0.8 to 1000 pg/mL and a remarkable LOD of 0.64 pg/mL.

### 4.3. Integration of Artificial Intelligence (AI) and Internet of Things (IoT)

The integration of AI and IoT technologies with electrochemical sensors has opened new avenues for enhancing sensitivity, selectivity, and real-time monitoring capabilities. While these advancements have not yet been widely applied to electrochemical immunosensors for OP analysis specifically, their potential impact on the field is significant and warrants discussion. AI, particularly machine learning algorithms, has shown promise in improving the performance of electrochemical sensors. For instance, Ye et al. [87] developed a deep learning model to analyze voltammetric data from an electrochemical sensor for heavy metal detection. Their convolutional neural network (CNN) approach significantly improved the sensor’s ability to distinguish between different metal ions in complex environmental samples, demonstrating how AI can enhance selectivity in electrochemical sensing. In the realm of pesticide detection, although not specifically for OPs, Gómez et al. [88] applied a support vector machine (SVM) algorithm to process data from an electrochemical sensor array for multiple pesticide residues. The AI-assisted data analysis allowed for the simultaneous quantification of several pesticides with overlapping electrochemical signatures, showcasing the potential for improved multi-analyte detection in complex matrices.

IoT integration has also shown potential in expanding the capabilities of electrochemical sensing systems. Ozer et al. [89] developed an IoT-enabled wearable device for monitoring potassium ions in sweat, aimed at point-of-care applications. The IoT was utilized to enable real-time data acquisition and transmission from the wearable device to a smartphone application. The device incorporated an Arduino-supported, Wi-Fi embedded microcontroller, specifically the ESP32, which facilitated wireless communication. This setup allowed the device to transmit potentiometric data via Wi-Fi to the smartphone app, where the data could be processed and displayed on an OLED screen.

## 5. Challenges and Future Perspectives

Table 1 summarizes the sensing performance of electrochemical immunosensors for OP detection. Despite significant advancements in electrochemical immunosensors for organophosphate detection, several challenges persist in real sample analysis [9,54]. Matrix effects from complex environmental and biological samples often interfere with sensor performance, leading to reduced sensitivity and selectivity [55,90]. Non-specific adsorption of interfering compounds on electrode surfaces can result in false positives or negatives [91,92,93]. Additionally, the stability of biological recognition elements, such as antibodies, in harsh environmental conditions remains a concern, potentially limiting the shelf life and reliability of these sensors in field applications.

Emerging trends in electrochemical immunosensors for organophosphate detection focus on overcoming these limitations. The integration of artificial intelligence and machine learning algorithms for data analysis and interpretation is gaining traction, enabling more accurate and reliable detection in complex matrices [94,95,96]. Nanomaterial-based aptasensors are emerging as alternatives to traditional antibody-based systems, offering improved stability and the potential for regeneration [97,98,99].

The potential for commercialization of electrochemical immunosensors for organophosphate detection is significant, driven by the growing demand for rapid, on-site monitoring tools in agriculture, environmental protection, and public health. Portable, user-friendly devices that integrate sample preparation, analysis, and data interpretation are particularly promising for field applications. However, challenges in scaling up production, ensuring long-term stability, and meeting regulatory requirements need to be addressed. Collaboration between academic researchers, industry partners, and regulatory agencies will be crucial in translating laboratory prototypes into commercially viable products.

**Figure 13:**
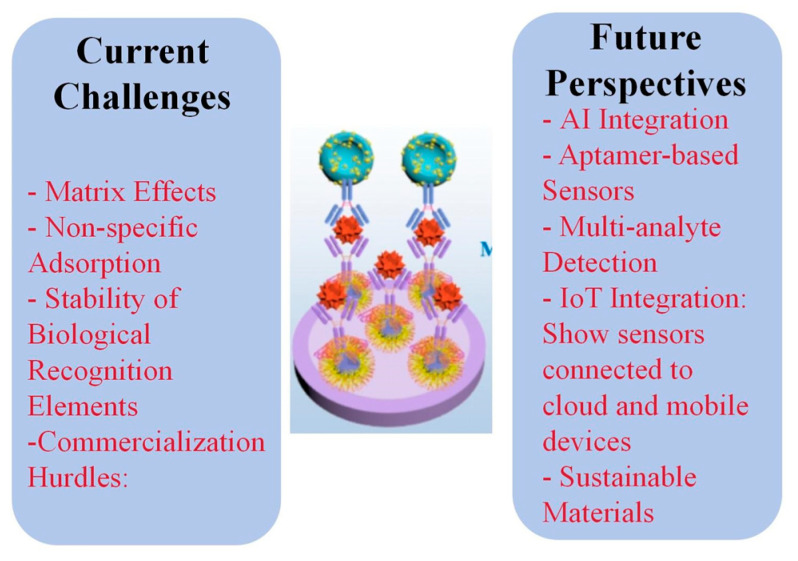
Infographic summarizing the current challenges and future perspectives of electrochemical immunosensors for OP analysis.

Future research in this field should focus on developing more robust and versatile sensing platforms capable of detecting multiple organophosphates simultaneously in diverse environmental and biological samples. Efforts to improve the stability and shelf life of biological recognition elements, possibly through the use of synthetic alternatives or encapsulation technologies, will be crucial. The exploration of novel nanomaterials and hybrid nanocomposites for signal amplification and improved electron transfer kinetics remains an active area of investigation. Additionally, research into sustainable and eco-friendly materials for sensor fabrication aligns with growing environmental concerns. Integrating these advanced sensors with emerging technologies such as IoT and cloud-based data analytics could pave the way for widespread, networked environmental monitoring systems. Figure 13 shows an infographic summarizing the current challenges and future perspectives discussed in this section.

**Table 1:** Sensing performance of electrochemical immunosensors for OP detection.

Sensor	Analyte	LDR	LOD	Real Sample	Ref.
GCE/SWNTs/PEG/FDMA/paraoxon hapten/anti-paraoxon IgG	Paraoxon	2 to 2500 ppb	2 ppb	Field water, lake water, tap water, and purified water	[29]
GCE/AuNPs/paraoxon antibodies	Paraoxon	24 to 1920 mg/L	12 mg/L	River water samples	[100]
Au-SPEs/SAM/AChE	Paraoxon	Up to 40 ppb	2 ppb	Drinking water samples and a sample from the Sacco River	[35]
GCE/SiSG/anti-carbofuran antibody	Carbofuran	1 ng/mL to 100 μg/mL and from 50 μg/mL to 200 μg/mL	0.33 ng/mL	Cabbage and lettuce	[101]
GCE/DpAu/PB-MWCNTs-CTS/Protein A	Carbofuran	0.1 ng/mL to 1 µg/mL	0.021 ng/mL	Cabbage and lettuce	[102]
GCE/MWCNTs/GS-PEI-Au/AuNPs-Ab	Carbofuran	0.5 to 500 ng/mL	0.03 ng/mL	Cabbages, green peppers, tomatoes, Chinese chives, and peaches	[103]
Au/L-Cys/GA/carbofuran antibodies	Carbofuran	0.1 to 1000 ng/mL	0.1 ng/mL	Tomato, cabbage, lettuce, soil, and water	[104]
Au/L-Cys/GNPs/anti-carbofuran/HRP	Carbofuran	0.01 ng/mL to 50 ng/mL	0.01 ng/mL	Cabbage and lettuce	[105]
Au/DpAu/Protein A/carbofuran antibodies	Carbofuran	1 to 100 ng/mL and 100 ng/mL to 100 μg/mL	0.1924 ng/mL	Chinese chives and celery cabbage	[106]
GCE/GNPs/Fe_3_O_4_-FCNTs-CS/anti-carbofuran antibody	Carbofuran	1.0 ng/mL to 100.0 ng/mL and 100.0 ng/mL to 200 μg/mL	0.032 ng/mL	Cabbage	[107]
SPE/HRP/atrazine antibody/Biodyne C membranes	Atrazine	-	0.012 mg/L	Water samples from the Yarqon River in Israel	[72]
SPE/AuNP/mAb	IMD	50 to 10,000 pM	22 pM	Tap water and watermelon samples	[85]
SPE/citric acid-decorated nylon nanofibers/Nb-ALP	3-Phenoxybenzoic acid	0.8 to 1000 pg/mL	0.64 pg/mL	Human urine samples	[86]
GCE/SWCNTs/antibodies	Endosulfan and paraoxon	2 to 2500 ppb.	0.05 ppb	Environmental water samples	[62]
SPE/graphene/2-ABA/anti-parathion antibodies	Parathion	0.1 to 1000 ng/L	52 pg/L	Tomato and carrot samples	[67]
SPE/GQDs/anti-parathion antibodies	Parathion	0.1 to 10^6^ ng/L	46 pg/L	Environmental and food samples	[68]
GCE/3D Au nanoclusters/BSA–picloram	Picloram	0.001 to 10 µg/mL	0.0005 µg/mL	Peach and excess sludge supernatant samples	[74]
FTO/GO/monocrotophos antibodies	Monocrotophos	-	0.49 ppm	Vegetable extracts and pond water	[69]
SPE/AuNP-Abs/PB	OPs (not specific)	1.82 × 10^−3^ to 3.29 × 10^4^ ng/mL	0.003 ng/mL	Baby cabbages and spinach	[30]
GCE/NBCQDs@GO/mAb3C9 antibody/phage-mimotope M31/anti-M13 mAb-HRP	O,O-dimethyl OPs	0.005 to 500 ng/mL	0.003–0.014 ng/mL for 9 O,O-dimethyl OPs	Cucumber, cabbage, and lettuce	[36]
GCE/AuNPs/PANI/MWCNTs/CS/anti-CPF antibody	CPF	-	-	Fruits and vegetables	[31]
Ab/AuNP/HRP/GCE	CPF	0.001 to 10 ng/mL	0.070 pg/mL	Chinese cabbage and lettuce	[71]
GCE/Q-PDANSs/anti-CPF antibody/multi-HRP-CNTs@f-Fe_3_O_4_-Ab2	CPF	0.01 to 1000 ng/mL	6.3 pg/mL	Pond water from farmland in South China Agricultural University	[47]
GraFET/Cr/Au	CPF	1 fM to 1 µM	1.8 fM	-	[81]
IDAM/PDDA/AuNPs/Protein A/anti-CPF	CPF	0.5 to 500 ng/mL	0.5 ng/mL	Cucumber, lettuce, and pakchoi	[80]
ITO/Co_3_O_4_/PAn/CPF-BSA antigen and CPF monoclonal antibodies	CPF	0 to 10 μg/mL	0.01 μg/mL	Green vegetables and apples	[59]
FTO-AuNPs-chl-Ab	CPF	1 fM to 1 μM	10 fM	Apple, cabbage, and pomegranate	[56]
SPE/PVA/G-AuNPs NFM/Nb8F	Quinalphos	0.06 to 1000 ng/mL	50.74 pg/mL	Lettuce and cucumber	[42]
GCE/chitosan/glutaraldehyde/fenvalerate monoclonal antibodies	Fenvalerate	1.0 × 10^−3^ to 1.0 × 10^−1^ mg/L	0.8 μg/L	Tea samples	[37]
SPE/PVA/CA NFM/VHH9-HRP	Parathion	0.01 to 100 ng/mL	2.26 pg/mL	Cucumber, orange, and Chinese cabbage	[43]
SPE/anti-TCP mouse antibody/HRP conjugated to HTCP	TCP	0.1 to 100 ng/mL	0.1 ng/mL	Rat plasma samples from rats exposed to CPF-oxon in vivo	[44]
GGE/Hap-Car-BSA@CuNPs conjugate	Carbaryl	0.8 to 32.3 μg/kg	0.08 μg/kg	Wheat, corn, and oats	[57]
SPE/ZrO_2_ NPs/Anti-BChE antibody/QDs	OP-BChE	0.1 to 30 nM	0.03 nM	Human plasma samples	[45]
SPE/Fe_3_O_4_@TiO_2_/QDs-anti-BChE	OP-BChE	0.02 to 10 nM	0.01 nM	Human plasma samples	[60]
SPE/ZrO_2_ NPs/LPA-anti-AChE	OP-AChE	0.05 to 10 nM	0.02 nM	Rat plasma samples	[58]
SPE/MWCNTs-Au/anti-AChE antibody	OP-AChE	0.2 to 50 nM	0.05 nM	Paraoxon-dosed red blood cell samples	[63]
SPE/ZrO_2_ NPs/QDs/anti-AChE antibody	OP-AChE	10 pM to 4 nM	8.0 pM	Human plasma	[108]

## 6. Conclusions

This review has explored the current trends and advancements in enhancing the sensitivity and selectivity of electrochemical immunosensors for organophosphate pesticide detection. The development of these sensors has been driven by the need for rapid, sensitive, and on-site monitoring tools to address the widespread use of organophosphates and their potential health and environmental impacts. Significant progress has been made in improving sensor performance through various strategies, including nanomaterial-based signal amplification, enzyme-based amplification cascades, and innovative electrode modifications. The principles and techniques discussed in this review, while focused on organophosphate detection, have broad implications for the field of electrochemical immunosensing as a whole. The strategies employed for enhancing sensitivity and selectivity in organophosphate detection can be adapted and applied to a wide range of target analytes. For instance, the use of metal nanoparticles and carbon nanomaterials for signal amplification, as demonstrated in organophosphate sensors, can be equally effective for detecting other environmental contaminants, biomarkers, and food safety targets. The innovative electrode modifications and three-dimensional architectures explored here offer promising approaches for improving the performance of electrochemical immunosensors across various applications.

The integration of these sensors into novel platforms such as microfluidic devices and lab-on-a-chip systems has opened up new possibilities for automated, high-throughput analysis. These advancements in miniaturization and integration are not limited to organophosphate detection but represent a broader trend in the development of portable, user-friendly diagnostic tools. Similarly, the use of screen-printed and disposable electrodes as cost-effective and versatile platforms for field applications can be extended to numerous other sensing scenarios. Despite these advancements, challenges remain in real sample analysis, including matrix effects and the stability of biological recognition elements in harsh environmental conditions. These challenges are common across many areas of electrochemical immunosensing and highlight the need for continued research and innovation in the field. The emerging trends discussed, such as the integration of artificial intelligence for data analysis and the development of aptamer-based sensors, offer promising solutions that could benefit electrochemical immunosensors for a variety of target analytes. The potential for commercialization of these sensors extends beyond organophosphate detection. The demand for rapid, on-site monitoring tools in agriculture, environmental protection, and public health is driving the development of versatile sensing platforms that can be adapted for multiple analytes. The lessons learned from developing organophosphate immunosensors can inform the design and optimization of sensors for other targets, potentially accelerating progress across the field.

Future research should focus on developing more robust and versatile sensing platforms capable of multi-analyte detection in diverse environmental and biological samples. Efforts to improve the stability and shelf life of biological recognition elements, possibly through the use of synthetic alternatives or encapsulation technologies, will be crucial for advancing not just organophosphate detection but electrochemical immunosensing as a whole. The exploration of novel nanomaterials and hybrid nanocomposites for signal amplification and improved electron transfer kinetics remains an active area of investigation with broad implications.

Additionally, research into sustainable and eco-friendly materials for sensor fabrication aligns with growing environmental concerns and could lead to more widely applicable, environmentally responsible sensing technologies. Integrating these advanced sensors with emerging technologies such as the Internet of Things (IoT) and cloud-based data analytics could pave the way for widespread, networked monitoring systems capable of detecting a range of analytes in real time. While this review has focused on organophosphate detection, the principles, techniques, and challenges discussed here are broadly applicable to the field of electrochemical immunosensing. The advancements made in organophosphate sensor development provide valuable insights and approaches that can be leveraged to enhance the sensitivity, selectivity, and practicality of electrochemical immunosensors for a wide range of applications. As the field continues to evolve, the cross-pollination of ideas and techniques across different sensing applications will be crucial in driving innovation and addressing complex analytical challenges in environmental monitoring, healthcare diagnostics, and beyond.

## Data Availability

No new data were created or analyzed in this study. Data sharing is not applicable to this article.

## References

[1] Karbelkar A.A., Reynolds E.E., Ahlmark R., Furst A.L. (2021). A Microbial Electrochemical Technology to Detect and Degrade Organophosphate Pesticides. ACS Cent. Sci..

[2] Kumaran A., Vashishth R., Singh S., Surendran U., James A., Chellam P.V. (2022). Biosensors for Detection of Organophosphate Pesticides: Current Technologies and Future Directives. Microchem. J..

[3] Patel H., Rawtani D., Agrawal Y.K. (2019). A Newly Emerging Trend of Chitosan-Based Sensing Platform for the Organophosphate Pesticide Detection Using Acetylcholinesterase—A Review. Trends Food Sci. Technol..

[4] Ayivi R.D., Obare S.O., Wei J. (2023). Molecularly Imprinted Polymers as Chemosensors for Organophosphate Pesticide Detection and Environmental Applications. TrAC Trends Anal. Chem..

[5] Bhattu M., Verma M., Kathuria D. (2021). Recent Advancements in the Detection of Organophosphate Pesticides: A Review. Anal. Methods.

[6] Cacho J.I., Campillo N., Viñas P., Hernández-Córdoba M. (2018). In Situ Ionic Liquid Dispersive Liquid-Liquid Microextraction Coupled to Gas Chromatography-Mass Spectrometry for the Determination of Organophosphorus Pesticides. J. Chromatogr. A.

[7] Rösch A., Beck B., Hollender J., Singer H. (2019). Picogram per Liter Quantification of Pyrethroid and Organophosphate Insecticides in Surface Waters: A Result of Large Enrichment with Liquid–Liquid Extraction and Gas Chromatography Coupled to Mass Spectrometry Using Atmospheric Pressure Chemical Ionization. Anal. Bioanal. Chem..

[8] Kaur R., Kaur R., Rani S., Malik A.K., Kabir A., Furton K.G. (2019). Application of Fabric Phase Sorptive Extraction with Gas Chromatography and Mass Spectrometry for the Determination of Organophosphorus Pesticides in Selected Vegetable Samples. J. Sep. Sci..

[9] Mollarasouli F., Kurbanoglu S., Ozkan S.A. (2019). The Role of Electrochemical Immunosensors in Clinical Analysis. Biosensors.

[10] Felix F.S., Angnes L. (2018). Electrochemical Immunosensors—A Powerful Tool for Analytical Applications. Biosens. Bioelectron..

[11] Kim J., Park M. (2021). Recent Progress in Electrochemical Immunosensors. Biosensors.

[12] Chen H., Zhang J., Huang R., Wang D., Deng D., Zhang Q., Luo L. (2023). The Applications of Electrochemical Immunosensors in the Detection of Disease Biomarkers: A Review. Molecules.

[13] Kumaravel A., Aishwarya S., Sathyamoorthi S. (2024). Emerging Technologies for Sensitive Detection of Organophosphate Pesticides: A Review. Curr. Anal. Chem..

[14] Chaudhari P., Chau L.-K., Ngo L.T., Chang T.-C., Chen Y.-L., Huang K.-T. (2023). Competitive Assay for the Ultrasensitive Detection of Organophosphate Pesticides Based on a Fiber-Optic Particle Plasmon Resonance Biosensor and an Acetylcholinesterase Binding Peptide. Anal. Chem..

[15] Arsawiset S., Sansenya S., Teepoo S. (2023). Nanozymes Paper−based Analytical Device for the Detection of Organophosphate Pesticides in Fruits and Vegetables. Anal. Chim. Acta.

[16] Li Y., Huang Z., Liu B., Huang Z.-Z., Yang H., Tan H. (2023). Portable Hydrogel Test Kit Integrated Dual-Emission Coordination Polymer Nanocomposite for on-Site Detection of Organophosphate Pesticides. Biosens. Bioelectron..

[17] Cho I.-H., Lee J., Kim J., Kang M., Paik J.K., Ku S., Cho H.-M., Irudayaraj J., Kim D.-H. (2018). Current Technologies of Electrochemical Immunosensors: Perspective on Signal Amplification. Sensors.

[18] Police Patil A.V., Chuang Y.-S., Li C., Wu C.-C. (2023). Recent Advances in Electrochemical Immunosensors with Nanomaterial Assistance for Signal Amplification. Biosensors.

[19] Kurup C.P., Mohd-Naim N.F., Ahmed M.U. (2022). Recent Trends in Nanomaterial-Based Signal Amplification in Electrochemical Aptasensors. Crit. Rev. Biotechnol..

[20] Zhang Y., Pan D., Zhou Q., Zhao J., Pan N., Zhang Y., Wang L., Shen Y. (2018). An Enzyme Cascade-Based Electrochemical Immunoassay Using a Polydopamine–Carbon Nanotube Nanocomposite for Signal Amplification. J. Mater. Chem. B.

[21] Cancelliere R., Paialunga E., Grattagliano A., Micheli L. (2024). Label-Free Electrochemical Immunosensors: A Practical Guide. TrAC Trends Anal. Chem..

[22] Turco A., Primiceri E., Chiriacò M.S., La Pesa V., Ferrara F., Riva N., Quattrini A., Romano A., Maruccio G. (2024). Advancing Amyotrophic Lateral Sclerosis Disease Diagnosis: A Lab-on-Chip Electrochemical Immunosensor for Ultra-Sensitive TDP-43 Protein Detection and Monitoring in Serum Patients’. Talanta.

[23] Felemban S., Vazquez P., Balbaied T., Moore E. (2022). Lab-on-a-Chip Electrochemical Immunosensor Array Integrated with Microfluidics: Development and Characterisation. Electrochem.

[24] Lopes P., Costa-Rama E., Beirão I., Nouws H.P.A., Santos-Silva A., Delerue-Matos C. (2019). Disposable Electrochemical Immunosensor for Analysis of Cystatin C, a CKD Biomarker. Talanta.

[25] Kämäräinen S., Mäki M., Tolonen T., Palleschi G., Virtanen V., Micheli L., Sesay A.M. (2018). Disposable Electrochemical Immunosensor for Cortisol Determination in Human Saliva. Talanta.

[26] Kabay G., Yin Y., Singh C.K., Ahmad N., Gunasekaran S., Mutlu M. (2022). Disposable Electrochemical Immunosensor for Prostate Cancer Detection. Sens. Actuators B Chem..

[27] Lee D., Bhardwaj J., Jang J. (2022). Paper-Based Electrochemical Immunosensor for Label-Free Detection of Multiple Avian Influenza Virus Antigens Using Flexible Screen-Printed Carbon Nanotube-Polydimethylsiloxane Electrodes. Sci. Rep..

[28] Abera B.D., Falco A., Ibba P., Cantarella G., Petti L., Lugli P. (2019). Development of Flexible Dispense-Printed Electrochemical Immunosensor for Aflatoxin M1 Detection in Milk. Sensors.

[29] Liu G., Song D., Chen F. (2013). Towards the Fabrication of a Label-Free Amperometric Immunosensor Using SWNTs for Direct Detection of Paraoxon. Talanta.

[30] Dong H., Zhao Q., Li J., Xiang Y., Liu H., Guo Y., Yang Q., Sun X. (2021). Broad-Spectrum Electrochemical Immunosensor Based on One-Step Electrodeposition of AuNP–Abs and Prussian Blue Nanocomposite for Organophosphorus Pesticide Detection. Bioprocess Biosyst. Eng..

[31] Ding J., Guo Y., Jia H., Qiao L., Sun X., Wang X. (2014). A Portable Pesticide Residues Detection Instrument Based on Impedance Immunosensor. Sens. Transducers.

[32] Qiao L., Jia H., Sun X., Wang X. (2014). Recent Advance of Electrochemical Immunosensor for Pesticide Residues Detection. Sens. Transducers.

[33] Diauudin F.N., Rashid J.I.A., Knight V.F., Wan Yunus W.M.Z., Ong K.K., Kasim N.A.M., Abdul Halim N., Noor S.A.M. (2019). A Review of Current Advances in the Detection of Organophosphorus Chemical Warfare Agents Based Biosensor Approaches. Sens. Bio-Sens. Res..

[34] Suri C.R., Raje M., Varshney G.C. (2002). Immunosensors for Pesticide Analysis: Antibody Production and Sensor Development. Crit. Rev. Biotechnol..

[35] Arduini F., Guidone S., Amine A., Palleschi G., Moscone D. (2013). Acetylcholinesterase Biosensor Based on Self-Assembled Monolayer-Modified Gold-Screen Printed Electrodes for Organophosphorus Insecticide Detection. Sens. Actuators B Chem..

[36] Shi R., Zou W., Zhao Z., Wang G., Guo M., Ai S., Zhou Q., Zhao F., Yang Z. (2022). Development of a Sensitive Phage-Mimotope and Horseradish Peroxidase Based Electrochemical Immunosensor for Detection of *O*,*O*-Dimethyl Organophosphorus Pesticides. Biosens. Bioelectron..

[37] Wang Z.-H., Viana A.S., Jin G., Abrantes L.M. (2006). Immunosensor Interface Based on Physical and Chemical Immunoglobulin G Adsorption onto Mixed Self-Assembled Monolayers. Bioelectrochemistry.

[38] Garcinuño B., Ojeda I., Moreno-Guzmán M., González-Cortés A., Yáñez-Sedeño P., Pingarrón J.M. (2014). Amperometric Immunosensor for the Determination of Ceruloplasmin in Human Serum and Urine Based on Covalent Binding to Carbon Nanotubes-Modified Screen-Printed Electrodes. Talanta.

[39] Li Y., Zhang Y., Jiang L., Chu P.K., Dong Y., Wei Q. (2016). A Sandwich-Type Electrochemical Immunosensor Based on the Biotin- Streptavidin-Biotin Structure for Detection of Human Immunoglobulin G. Sci. Rep..

[40] Patel M., Agrawal M., Srivastava A. (2022). Signal Amplification Strategies in Electrochemical Biosensors via Antibody Immobilization and Nanomaterial-Based Transducers. Mater. Adv..

[41] Radecka H., Radecki J. (2015). Label-Free Electrochemical Immunosensors for Viruses and Antibodies Detection-Review. J. Mex. Chem. Soc..

[42] Hu J., Wen P., Wang Y., Yang J., Xiao Z., Xu Z., Shen Y., Wang H., Hammock B.D. (2024). Fabrication of a Label-Free Electrochemical Immunosensor by Functionalized Nanofiber Membrane for the Ultrasensitive Detection of Quinalphos. Food Control.

[43] Yin W., Zhang J., Wang H., Wang Y., Zeng X., Xu Z., Yang J., Xiao Z., Hammock B.D., Wen P. (2023). A Highly Sensitive Electrochemical Immunosensor Based on Electrospun Nanocomposite for the Detection of Parathion. Food Chem..

[44] Wang L., Lu D., Wang J., Du D., Zou Z., Wang H., Smith J.N., Timchalk C., Liu F., Lin Y. (2011). A Novel Immunochromatographic Electrochemical Biosensor for Highly Sensitive and Selective Detection of Trichloropyridinol, a Biomarker of Exposure to Chlorpyrifos. Biosens. Bioelectron..

[45] Lu D., Wang J., Wang L., Du D., Timchalk C., Barry R., Lin Y. (2011). A Novel Nanoparticle-Based Disposable Electrochemical Immunosensor for Diagnosis of Exposure to Toxic Organophosphorus Agents. Adv. Funct. Mater..

[46] Wang H., Ma Z. (2018). A Cascade Reaction Signal-Amplified Amperometric Immunosensor Platform for Ultrasensitive Detection of Tumour Marker. Sens. Actuators B Chem..

[47] Sun Z., Wang W., Wen H., Gan C., Lei H., Liu Y. (2015). Sensitive Electrochemical Immunoassay for Chlorpyrifos by Using Flake-like Fe_3_O_4_ Modified Carbon Nanotubes as the Enhanced Multienzyme Label. Anal. Chim. Acta.

[48] Reynoso E.C., Torres E., Bettazzi F., Palchetti I. (2019). Trends and Perspectives in Immunosensors for Determination of Currently-Used Pesticides: The Case of Glyphosate, Organophosphates, and Neonicotinoids. Biosensors.

[49] Ding R., Li Z., Xiong Y., Wu W., Yang Q., Hou X. (2023). Electrochemical (Bio)Sensors for the Detection of Organophosphorus Pesticides Based on Nanomaterial-Modified Electrodes: A Review. Crit. Rev. Anal. Chem..

[50] Uniyal S., Sharma R.K. (2018). Technological Advancement in Electrochemical Biosensor Based Detection of Organophosphate Pesticide Chlorpyrifos in the Environment: A Review of Status and Prospects. Biosens. Bioelectron..

[51] Carneiro P., Loureiro J.A., Delerue-Matos C., Morais S., do Carmo Pereira M. (2023). Nanostructured Label–Free Electrochemical Immunosensor for Detection of a Parkinson’s Disease Biomarker. Talanta.

[52] Boonkaew S., Teengam P., Jampasa S., Rengpipat S., Siangproh W., Chailapakul O. (2020). Cost-Effective Paper-Based Electrochemical Immunosensor Using a Label-Free Assay for Sensitive Detection of Ferritin. Analyst.

[53] Surappa S., Multani P., Parlatan U., Sinawang P.D., Kaifi J., Akin D., Demirci U. (2023). Integrated “Lab-on-a-Chip” Microfluidic Systems for Isolation, Enrichment, and Analysis of Cancer Biomarkers. Lab Chip.

[54] Akbari Nakhjavani S., Afsharan H., Khalilzadeh B., Ghahremani M.H., Carrara S., Omidi Y. (2019). Gold and Silver Bio/Nano-Hybrids-Based Electrochemical Immunosensor for Ultrasensitive Detection of Carcinoembryonic Antigen. Biosens. Bioelectron..

[55] Yang G., Lai Y., Xiao Z., Tang C., Deng Y. (2018). Ultrasensitive Electrochemical Immunosensor of Carcinoembryonic Antigen Based on Gold-Label Silver-Stain Signal Amplification. Chin. Chem. Lett..

[56] Talan A., Mishra A., Eremin S.A., Narang J., Kumar A., Gandhi S. (2018). Ultrasensitive Electrochemical Immuno-Sensing Platform Based on Gold Nanoparticles Triggering Chlorpyrifos Detection in Fruits and Vegetables. Biosens. Bioelectron..

[57] Dorozhko E.V., Gashevskay A.S., Korotkova E.I., Barek J., Vyskocil V., Eremin S.A., Galunin E.V., Saqib M. (2021). A Copper Nanoparticle-Based Electrochemical Immunosensor for Carbaryl Detection. Talanta.

[58] Du D., Chen A., Xie Y., Zhang A., Lin Y. (2011). Nanoparticle-Based Immunosensor with Apoferritin Templated Metallic Phosphate Label for Quantification of Phosphorylated Acetylcholinesterase. Biosens. Bioelectron..

[59] Wang W., Han Z., Liang P., Guo D., Xiang Y., Tian M., Song Z., Zhao H. (2017). Co_3_O_4_/PAn Magnetic nanoparticle-modified electrochemical immunosensor for chlorpyrifos. Dig. J. Nanomater. Biostruct. (DJNB).

[60] Zhang X., Wang H., Yang C., Du D., Lin Y. (2013). Preparation, Characterization of Fe_3_O_4_ at TiO_2_ Magnetic Nanoparticles and Their Application for Immunoassay of Biomarker of Exposure to Organophosphorus Pesticides. Biosens. Bioelectron..

[61] Sobhan A., Jia F., Kelso L.C., Biswas S.K., Muthukumarappan K., Cao C., Wei L., Li Y. (2022). A Novel Activated Biochar-Based Immunosensor for Rapid Detection of *E. coli* O157:H7. Biosensors.

[62] Liu G., Guo W., Song D. (2014). A Multianalyte Electrochemical Immunosensor Based on Patterned Carbon Nanotubes Modified Substrates for Detection of Pesticides. Biosens. Bioelectron..

[63] Chen A., Du D., Lin Y. (2012). Highly Sensitive and Selective Immuno-Capture/Electrochemical Assay of Acetylcholinesterase Activity in Red Blood Cells: A Biomarker of Exposure to Organophosphorus Pesticides and Nerve Agents. Environ. Sci. Technol..

[64] Yang M., Javadi A., Li H., Gong S. (2010). Ultrasensitive Immunosensor for the Detection of Cancer Biomarker Based on Graphene Sheet. Biosens. Bioelectron..

[65] Li B., Tan H., Jenkins D., Srinivasa Raghavan V., Rosa B.G., Güder F., Pan G., Yeatman E., Sharp D.J. (2020). Clinical Detection of Neurodegenerative Blood Biomarkers Using Graphene Immunosensor. Carbon.

[66] Andoy N.M., Filipiak M.S., Vetter D., Gutiérrez-Sanz Ó., Tarasov A. (2018). Graphene-Based Electronic Immunosensor with Femtomolar Detection Limit in Whole Serum. Adv. Mater. Technol..

[67] Mehta J., Vinayak P., Tuteja S.K., Chhabra V.A., Bhardwaj N., Paul A.K., Kim K.-H., Deep A. (2016). Graphene Modified Screen Printed Immunosensor for Highly Sensitive Detection of Parathion. Biosens. Bioelectron..

[68] Mehta J., Bhardwaj N., Bhardwaj S.K., Tuteja S.K., Vinayak P., Paul A.K., Kim K.-H., Deep A. (2017). Graphene Quantum Dot Modified Screen Printed Immunosensor for the Determination of Parathion. Anal. Biochem..

[69] Shrikrishna N.S., Kolhe P., Gandhi S. (2024). Sensitive Detection of Monocrotophos Using a Voltametric Immunosensor with Randomly Layered Graphene Oxide (GO) on Fabricated Electrode. J. Environ. Chem. Eng..

[70] Cancelliere R., Cosio T., Campione E., Corvino M., D’Amico M.P., Micheli L., Signori E., Contini G. (2023). Label-Free Electrochemical Immunosensor as a Reliable Point-of-Care Device for the Detection of Interleukin-6 in Serum Samples from Patients with Psoriasis. Front. Chem..

[71] Hou L., Zhang X., Kong M., Jiang G., Sun Y., Mo W., Lin T., Ye F., Zhao S. (2020). A Competitive Immunoassay for Electrochemical Impedimetric Determination of Chlorpyrifos Using a Nanogold-Modified Glassy Carbon Electrode Based on Enzymatic Biocatalytic Precipitation. Microchim. Acta.

[72] Keay R.W., McNeil C.J. (1998). Separation-Free Electrochemical Immunosensor for Rapid Determination of Atrazine. Biosens. Bioelectron..

[73] Chansi, Bhardwaj R., Rao R.P., Mukherjee I., Agrawal P.K., Basu T., Bharadwaj L.M. (2020). Layered Construction of Nano Immuno-Hybrid Embedded MOF as an Electrochemical Sensor for Rapid Quantification of Total Pesticides Load in Vegetable Extract. J. Electroanal. Chem..

[74] Chen L., Zeng G., Zhang Y., Tang L., Huang D., Liu C., Pang Y., Luo J. (2010). Trace Detection of Picloram Using an Electrochemical Immunosensor Based on Three-Dimensional Gold Nanoclusters. Anal. Biochem..

[75] Rahmani T., Bagheri H., Behbahani M., Hajian A., Afkhami A. (2018). Modified 3D Graphene-Au as a Novel Sensing Layer for Direct and Sensitive Electrochemical Determination of Carbaryl Pesticide in Fruit, Vegetable, and Water Samples. Food Anal. Methods.

[76] Gokila N., Haldorai Y., Saravanan P., Rajendra Kumar R.T. (2024). Non-Enzymatic Electrochemical Impedance Sensor for Selective Detection of Electro-Inactive Organophosphate Pesticides Using Zr-MOF/ZrO2/MWCNT Ternary Composite. Environ. Res..

[77] Verma N., Pandya A., Pandya A., Singh V. (2022). Chapter Twelve—Challenges and Opportunities in Micro/Nanofluidic and Lab-on-a-Chip. Progress in Molecular Biology and Translational Science.

[78] Azizipour N., Avazpour R., Rosenzweig D.H., Sawan M., Ajji A. (2020). Evolution of Biochip Technology: A Review from Lab-on-a-Chip to Organ-on-a-Chip. Micromachines.

[79] Gurkan U.A., Wood D.K., Carranza D., Herbertson L.H., Diamond S.L., Du E., Guha S., Di Paola J., Hines P.C., Papautsky I. (2024). Next Generation Microfluidics: Fulfilling the Promise of Lab-on-a-Chip Technologies. Lab Chip.

[80] Jia H., Guo Y., Sun X., Wang X. (2015). An Electrochemical Immunosensor Based on Microfluidic Chip for Detection of Chlorpyrifos. Int. J. Electrochem. Sci..

[81] Islam S., Shukla S., Bajpai V.K., Han Y.-K., Huh Y.S., Ghosh A., Gandhi S. (2019). Microfluidic-Based Graphene Field Effect Transistor for Femtomolar Detection of Chlorpyrifos. Sci. Rep..

[82] García-Miranda Ferrari A., Rowley-Neale S.J., Banks C.E. (2021). Screen-Printed Electrodes: Transitioning the Laboratory in-to-the Field. Talanta Open.

[83] Costa-Rama E., Fernández-Abedul M.T. (2021). Paper-Based Screen-Printed Electrodes: A New Generation of Low-Cost Electroanalytical Platforms. Biosensors.

[84] Martínez-Periñán E., Gutiérrez-Sánchez C., García-Mendiola T., Lorenzo E. (2020). Electrochemiluminescence Biosensors Using Screen-Printed Electrodes. Biosensors.

[85] Pérez-Fernández B., Mercader J.V., Abad-Fuentes A., Checa-Orrego B.I., Costa-García A., de la Escosura-Muñiz A. (2020). Direct Competitive Immunosensor for Imidacloprid Pesticide Detection on Gold Nanoparticle-Modified Electrodes. Talanta.

[86] El-Moghazy A.Y., Huo J., Amaly N., Vasylieva N., Hammock B.D., Sun G. (2020). An Innovative Nanobody-Based Electrochemical Immunosensor Using Decorated Nylon Nanofibers for Point-of-Care Monitoring of Human Exposure to Pyrethroid Insecticides. ACS Appl. Mater. Interfaces.

[87] Ye J.-J., Lin C.-H., Huang X.-J. (2020). Analyzing the Anodic Stripping Square Wave Voltammetry of Heavy Metal Ions via Machine Learning: Information beyond a Single Voltammetric Peak. J. Electroanal. Chem..

[88] Gómez J.K.C., Puentes Y.A.N., Niño D.D.C., Acevedo C.M.D. (2023). Detection of Pesticides in Water through an Electronic Tongue and Data Processing Methods. Water.

[89] Ozer T., Agir I., Henry C.S. (2022). Low-Cost Internet of Things (IoT)-Enabled a Wireless Wearable Device for Detecting Potassium Ions at the Point of Care. Sens. Actuators B Chem..

[90] Fratus M., Alam M.A. (2023). Universal Scaling Theory of Electrochemical Immunosensors: An Analytical Approach to Define and Compare Performance Metrics. Appl. Phys. Lett..

[91] Martins B.R., Barbosa Y.O., Andrade C.M.R., Pereira L.Q., Simão G.F., de Oliveira C.J., Correia D., Oliveira R.T.S., da Silva M.V., Silva A.C.A. (2020). Development of an Electrochemical Immunosensor for Specific Detection of Visceral Leishmaniasis Using Gold-Modified Screen-Printed Carbon Electrodes. Biosensors.

[92] Zumpano R., Polli F., D’Agostino C., Antiochia R., Favero G., Mazzei F. (2021). Nanostructure-Based Electrochemical Immunosensors as Diagnostic Tools. Electrochem.

[93] Ochoa-Ruiz A.G., Parra G., López-Espinoza D., Astudillo P., Galyamin D., Sabaté N., Esquivel J.P., Vallejo-Cardona A.A. (2023). Electrochemical Immunosensors: The Evolution from Elisa to EμPADs. Electroanalysis.

[94] Magarelli G., Freire A.M., Silva L.P., Shukla A.K. (2023). Chapter Seven—Electrochemical Sensors Coupled with Machine Learning for Food Safety and Quality Inspection. Food Quality Analysis.

[95] Chakraborty K., Ebihara A. (2024). Pesticide Biosensors for Multiple Target Detection: Improvement Potential with Advanced Data-Processing Methods. Rev. Agric. Sci..

[96] Cui F., Yue Y., Zhang Y., Zhang Z., Zhou H.S. (2020). Advancing Biosensors with Machine Learning. ACS Sens..

[97] Phopin K., Tantimongcolwat T. (2020). Pesticide Aptasensors—State of the Art and Perspectives. Sensors.

[98] Yang F., Li J., Dong H., Wang G., Han J., Xu R., Kong Q., Huang J., Xiang Y., Yang Q. (2022). A Novel Label-Free Electrochemiluminescence Aptasensor Using a Tetrahedral DNA Nanostructure as a Scaffold for Ultrasensitive Detection of Organophosphorus Pesticides in a Luminol–H_2_O_2_ System. Analyst.

[99] Liu M., Khan A., Wang Z., Liu Y., Yang G., Deng Y., He N. (2019). Aptasensors for Pesticide Detection. Biosens. Bioelectron..

[100] Hu S.-Q., Xie J.-W., Xu Q.-H., Rong K.-T., Shen G.-L., Yu R.-Q. (2003). A Label-Free Electrochemical Immunosensor Based on Gold Nanoparticles for Detection of Paraoxon. Talanta.

[101] Sun X., Du S., Wang X., Zhao W., Li Q. (2011). A Label-Free Electrochemical Immunosensor for Carbofuran Detection Based on a Sol-Gel Entrapped Antibody. Sensors.

[102] Sun X., Du S., Wang X. (2012). Amperometric Immunosensor for Carbofuran Detection Based on Gold Nanoparticles and PB-MWCNTs-CTS Composite Film. Eur. Food Res. Technol..

[103] Zhu Y., Cao Y., Sun X., Wang X. (2013). Amperometric Immunosensor for Carbofuran Detection Based on MWCNTs/GS-PEI-Au and AuNPs-Antibody Conjugate. Sensors.

[104] Liu L., Xu D., Hu Y., Liu S., Wei H., Zheng J., Wang G., Hu X., Wang C. (2015). Construction of an Impedimetric Immunosensor for Label-Free Detecting Carbofuran Residual in Agricultural and Environmental Samples. Food Control.

[105] Du S., Wang X., Sun X., Li Q. (2012). Amperometric Immunosensor Based on L-Cysteine/Gold Colloidal Nanoparticles for Carbofuran Detection. Anal. Lett..

[106] Sun X., Zhu Y., Wang X. (2011). Amperometric Immunosensor Based on a Protein A/Deposited Gold Nanocrystals Modified Electrode for Carbofuran Detection. Sensors.

[107] Sun X., Li Q., Wang X., Du S. (2012). Amperometric Immunosensor Based on Gold Nanoparticles/Fe_3_O_4_-FCNTs-CS Composite Film Functionalized Interface for Carbofuran Detection. Anal. Lett..

[108] Liu G., Wang J., Barry R., Petersen C., Timchalk C., Gassman P.L., Lin Y. (2008). Nanoparticle-Based Electrochemical Immunosensor for the Detection of Phosphorylated Acetylcholinesterase: An Exposure Biomarker of Organophosphate Pesticides and Nerve Agents. Chem. Eur. J..

